# Linear Basal Cell Carcinoma in an Asian Patient

**DOI:** 10.2174/1874364100701010020

**Published:** 2007-12-17

**Authors:** Kinoshita Shinsuke, Kakizaki Hirohiko, Takahashi Yasuhiro, Hara Kazuo, Iwaki Masayoshi

**Affiliations:** 1 Department of Ophthalmology, Aichi Medical University, Nagakute, Aichi, 480-1195, Japan; 2 Department of Ophthalmology and Visual Sciences, Osaka City University Graduate School of Medicine, 1-4-3, Asahi-machi, Abeno-ku, Osaka, 545-8585, Japan; 3 Department of Pathology, Aichi Medical University Hospital, Nagakute, Aichi, 480-1195, Japan

## Abstract

Linear basal cell carcinoma (BCC), which has a ratio of its long and short axes of more than 3: 1, is a distinct clinical entity among BCC. We report the first case report of a linear BCC in an Asian patient. An 87 year-old woman presented with an ulcerated black nodule, 15×5mm (3: 1), on her nasojungal fold of the right lower eyelid. The tumor was excised with 5 mm safety margin. The pathological examination confirmed the tumor was a BCC with a clear margin. Diagnosis of a linear BCC is based on its morphological features and occurrence along the wrinkle line, which needs to be also considered in Asian.

## INTRODUCTION

Linear basal cell carcinoma (BCC), first reported in 1985, is a definite clinical entity among BCC [1]. It shows a linear form along a wrinkle line [2] and the proportion of its long and short axes is more than 3: 1 [3,4]. The most usual sites for it to occur are the lower eyelid, cheek and neck areas [1,3-6]. Although 33 cases have been reported so far, to our knowledge, there are no reports of its occurrence in Asians [1,3-6].

Most linear BCC microscopically show a “nodular” subtype, and have low recurrence [3,4,6], which is similar to BCC in general [8-10]. However, 25 to 40% of linear BCC demonstrate high risk subtypes with a high recurrence rates, such as micronodular, infiltrative and morpheic subtypes [3,4,7]. Since only 6% of all BCC are high risk types, linear BCC has a high possibility of being a high risk subtype [7]. This is a reason that linear BCC is judged as a definite clinical entity among BCC [3,4]. Therefore, meticulous care needs to be taken with linear BCC during follow up periods [7,8].

We here report a case of a linear BCC that occurred on the nasojugal fold of the right lower eyelid. This is the first case report of a linear BCC involving an Asian patient.

## CASE REPORT

**Fig. (1):**
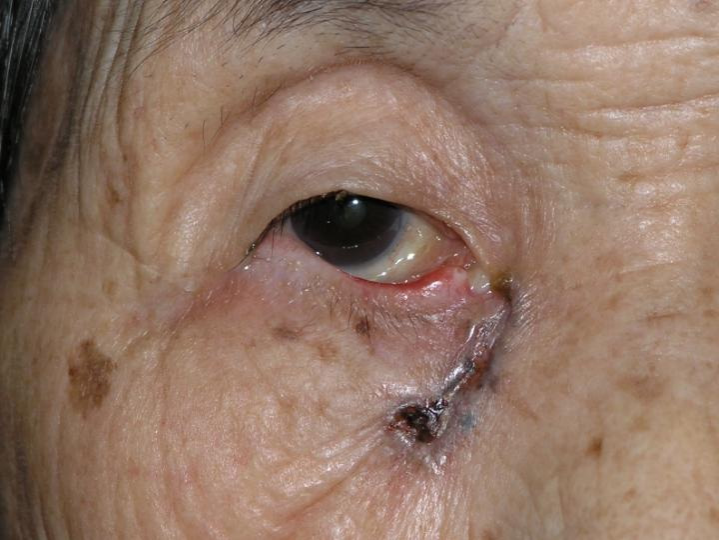
A black ulcerated nodule, 15 x 5 mm (3: 1), can be seen on the nasojungal fold of the right lower eyelid. The lower eyelid shows medial ectropion and the lateral margin of it is situated to around the half of the lower eyelid.

**Fig. (2):**
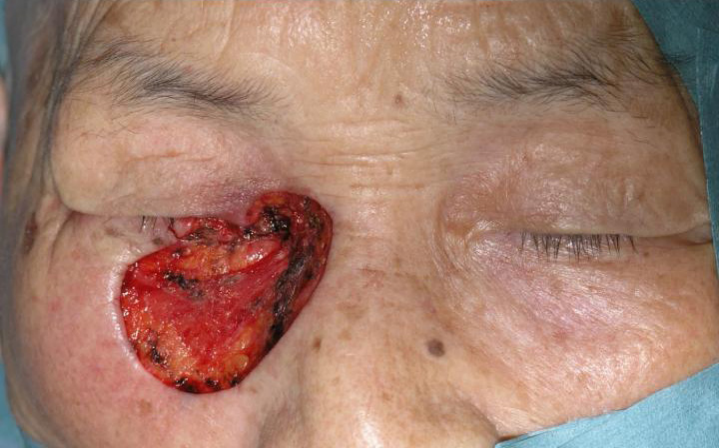
The linear basal cell carcinoma was excised with 5 mm safety margin. As the skin had been pulled toward the tumour, when the traction was released, the defect became very large.

**Fig. (3):**
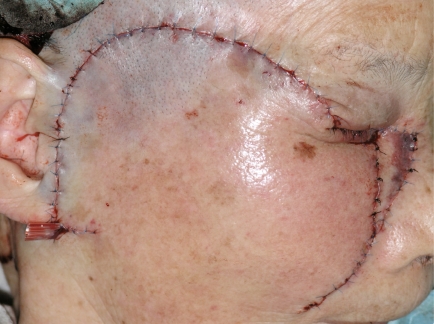
The defect was reconstructed with a cheek rotation flap for the lower eyelid area and a nasolabial VY advancement flap for the medial canthal area.

**Fig. (4):**
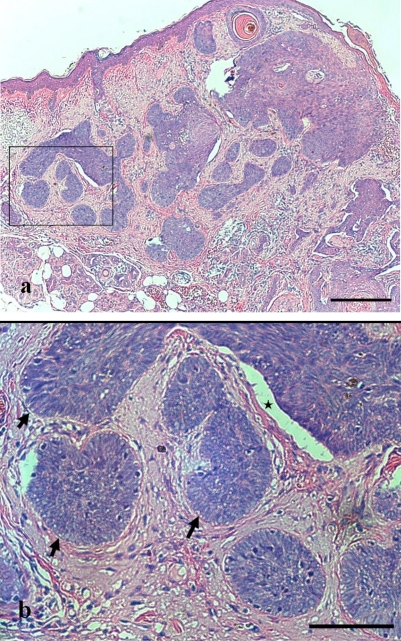
Pathological specimen (Hematoxylin and Eosin). Typical “nodular” subtype is demonstrated. **a.** Tiny tumor islands are demonstrated. (bar = 200 μm) **b.** The magnification of the rectangle in a. Palisading (allow) and clefting (star) are shown. (bar = 100 μm).

**Fig. (5):**
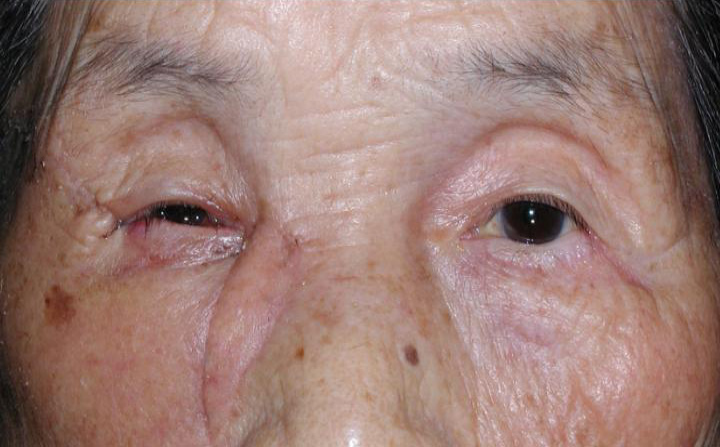
Postoperative findings show no local recurrence or lower eyelid ectropion. By reconstructing the lower eyelid suspending laterally, the lower eyelid slope toward nasally was made to flow the lacrimal fluid.

An 87 year-old woman presented with an ulcerated black nodule, 15 x 5 mm (3: 1), on nasojugal fold of the right lower eyelid (Fig. **1**). Her lower eyelid was everted toward the tumour because of the traction and the lateral margin of the tumour was situated to around the half of the lower eyelid. Computed tomography did not show any spread to deep orbital structures or to bone. Based on these clinical findings, a linear BCC was suspected. The tumour was excised with a 5 mm safety margin, and the traction of the skin was completely released, after which the defect of the tumour became very large (Fig. **2**). The defect was reconstructed with a cheek rotation flap for the lower eyelid area and a nasolabial VY advancement flap for the medial canthal area (Fig. **3**). The upper lacrimal outflow system could be left, and by reconstructing the lower eyelid suspending laterally, the lower eyelid slope toward nasally was made to flow the lacrimal fluid. The pathological diagnosis was “BCC (nodular type)” with a clear margin (Fig. **4**). No perineural and vascular invasion was noted. Postoperatively, although the patient felt a little epiphora, its extent was not so severe. No recurrence or metastasis was found within the following 12 months (Fig. **5**).

## DISCUSSION

We have reported the first case of a linear BCC in an Asian patient. In general, malignant skin tumours are well seen in White patients with a lot of sun exposure [11]. On the other hand in Asians, occurrence of the malignant skin tumours is much less than the White population [11]. Therefore, it is important to understand that Asians can take a same entity.

BCC is the most common human malignancy and macroscopically can be divided into 3 main subtypes: noduloulcerative, superficial and sclerosing (morpheaform) [1]. Since these 3 subtypes have various appearances, only 60 to 70% of clinical diagnoses correspond with the pathological diagnosis [12]. However, as linear BCC show a peculiar linear form along a wrinkle line [1,3-6], confident clinical diagnosis is possible. The present tumour clinically demonstrated a typical noduloulcerative subtype and occurred along the nasojugal wrinkle line, which enabled us to diagnose it easily.

The first choice therapy for BCC is surgical excision [13]; the same for linear BCC [1,3-6]. Here, we established a safety margin of 5 mm and so obtained a clear margin. This was based on evidence that 95% of BCC, except for the sclerosing (morpheaform) subtype, of less than 20 mm diameter show a clear margin if there is a safety margin of 5 mm [12]. Although the frozen section control and the Mohs’ micrographic surgery are the most common procedure for excision of a BCC [11,14], a procedure with a safety margin of 5 mm also assures a low recurrent rate of 1.3% to 3% [15,16]. Standard 5 mm margins can lead to incomplete excision in a proportion of cases, but it is useful when the frozen section control or the Mohs’ micrographic surgery cannot be undertaken.

## CONCLUSIONS

This is the first report of a linear BCC in an Asian patient. Diagnosis of linear BCC is based on its morphological features and occurrence along the wrinkle line, which needs to be also considered in Asian.
